# Cytogenetic analysis of *HER1*/*EGFR*, *HER2*, *HER3 *and *HER4 *in 278 breast cancer patients

**DOI:** 10.1186/bcr1843

**Published:** 2008-01-08

**Authors:** Andrea Sassen, Justine Rochon, Peter Wild, Arndt Hartmann, Ferdinand Hofstaedter, Stephan Schwarz, Gero Brockhoff

**Affiliations:** 1 Institute of Pathology, University of Regensburg, Franz-Josef-Strauss-Allee 11, 93053 Regensburg, Germany; 2 Center for Clinical Studies, University of Regensburg, Franz-Josef-Strauss-Allee 11, 93053 Regensburg, Germany; 3 Institute of Pathology, University Hospital Zurich, Schmelzbergstrasse 12, 8091 Zurich, Switzerland; 4 Institute of Pathology, University of Erlangen, Krankenhausstrasse 12, 91054 Erlangen, Germany

## Abstract

**Introduction:**

The HER (human EGFR related) family of receptor tyrosine kinases (HER1/EGFR (epidermal growth factor receptor)/c-erbB1, HER2/c-erbB2, HER3/c-erbB3 and HER4/c-erbB4) shares a high degree of structural and functional homology. It constitutes a complex network, coupling various extracellular ligands to intracellular signal transduction pathways resulting in receptor interaction and cross-activation. The most famous family member is HER2, which is a target in Herceptin™ therapy in metastatic status and also in adjuvant therapy of breast cancer in the event of dysregulation as a result of gene amplification and resulting protein overexpression. The HER2-related HER receptors have been shown to interact directly with HER2 receptors and thereby mutually affect their activity and subsequent malignant growth potential. However, the clinical outcome with regard to total HER receptor state remains largely unknown.

**Methods:**

We investigated HER1–HER4, at both the DNA and the protein level, using fluorescence *in situ *hybridisation (FISH) probes targeted to all four receptor loci and also immunohistochemistry in tissue microarrays derived from 278 breast cancer patients.

**Results:**

We retrospectively found *HER3 *gene amplification with a univariate negative impact on disease-free survival (hazard ratio 2.35, 95% confidence interval 1.08 to 5.11, *p *= 0.031), whereas *HER4 *amplification showed a positive trend in overall and disease-free survival. Protein expression revealed no additional information.

**Conclusion:**

Overall, the simultaneous quantification of *HER3 *and *HER4 *receptor genes by means of FISH might enable the rendering of a more precise stratification of breast cancer patients by providing additional prognostic information. The continuation of explorative and prospective studies on all HER receptors will be required for an evaluation of their potential use for specific therapeutic targeting with respect to individualised therapy.

## Introduction

Gene amplification of *HER2 *(*HER2*/*neu*, *c-erbB2*) receptor tyrosine kinase (RTK) is found in 10 to 25% of invasive breast carcinomas [1,2] and is associated with an unfavourable impact on the course of disease and reduced responsiveness to tamoxifen therapy, for example [3,4]. The HER2 receptor has frequently been described as dominantly triggering mitogenic signalling within the type 1 growth factor receptor family. As a ligandless orphan receptor, HER2 preferentially heterodimerises with its relatives [5,6] and thereby has an important role in signal triggering and amplification. Its malignant potential and its key role in enhanced cell proliferation, carcinogenesis, tumour progression and metastasis have frequently been proved in numerous preclinical and clinical studies [7].

The overexpressed receptor protein is exploited as the therapeutic target for Herceptin™, known as the humanised monoclonal antibody trastuzumab, in metastatic breast cancer and has recently proved useful in designing adjuvant treatment for breast carcinoma [8]. Moreover, strong HER2 expression represents the decisive molecular basis for tumour therapy targeted at the same receptor. However, a therapeutic benefit in terms of tumour regression, prolongation of recurrence-free survival and even overall survival [9] is found for about 50% of patients [9-11] depending on previous therapies, antibody resistance and combination with other chemotherapeutics such as paclitaxel or docetaxel [12]. This observation reflects the substantial insufficiency of using *HER2 *gene amplification or HER2 protein overexpression to predict patient responsiveness to Herceptin.

Hence, the identification of clinicopathological and molecular characteristics of breast cancer to enable more accurate prognosis of the course of disease and prediction of therapy response to antibodies or small enzyme-inhibiting molecules [13-15], for example, is a continuing challenge in the field of diagnostic pathology. To this end, the three additional members of the HER (human EGFR related)–RTK family HER1 (epidermal growth factor receptor (EGFR), c-erbB1), HER3 (c-erbB3) and HER4 (c-erbB4) are of particular interest because of their ability to interact directly with HER2 [16]. On the basis of their common evolutionary origin these receptors share a high degree of structural and functional homology, which is the molecular basis for receptor interaction and cross-activation [17]. Thus, HER-receptor activity and functionality depend on one another and thus the impact on tumour cell proliferation and growth is likely to be dependent on HER-receptor coexpression and communication.

Several immunohistochemical studies have been undertaken to elucidate the coexpression profile of HER receptors in breast cancer, providing preliminary data on other HER receptors besides HER2, which may have an impact on the course of disease and therapy responsiveness in breast cancer patients [18-20].

In this study we performed a four-target fluorescence *in situ *hybridisation (FISH) analysis of the *HER1*, *HER2*, *HER3 *and *HER4 *gene loci together with centromere quantification using 278 primary breast cancer samples compiled into a tissue microarray (TMA). Additionally, we immunohistochemically stained the receptor proteins and categorised staining intensity in accordance with EGFR pharmDX™ and HercepTest™ scoring guidelines. Furthermore, the results were compared with the Ki-67 proliferation index, a prognostic marker in early breast cancer [21]. Our objective was to determine the potential association between *HER1*-*HER4 *gene amplification or altered protein expression and outcome and course of disease, as well as with known clinicopathological breast cancer prognosticators [22]. We addressed the question of whether alteration in the *HER1*, *HER3 *or *HER4 *genes or their protein products conveys any prognostic value that is complementary to or independent of HER2 that would allow a more precise rendering of breast cancer patients into subgroups with different clinical outcomes based on HER-receptor analysis.

Our data indicate additional *HER3 *and *HER4 *prognostic markers in breast cancer that should be prospectively explored in further detail. The integration of *HER3 *and *HER4 *analysis into routine cancer diagnosis would provide valuable additional information. Further descriptive and particularly functional studies are required to understand their impact on the course of disease at the molecular and cellular levels [16,23] and will provide the basis for designing specific targeted therapeutics in terms of individualised disease management.

## Materials and methods

This study was approved by the Institutional Review Board of the University of Regensburg, Germany.

### Breast tumour samples and patient characteristics

Formalin-fixed paraffin-embedded tissue blocks from 278 female patients with invasive lobular or ductal unilateral primary breast cancer (median age 55 years; range 25 to 82 years) were obtained from the archives of the Institute of Pathology, Regensburg, Germany, and were derived from a consecutive series of sporadic breast cancers. The patients were not involved in any clinical trial. Clinical data were acquired by the Tumour Centre Inc., Regensburg. All patients underwent surgery between 1992 and 2002. The histopathological characteristics are listed in Table 1. The median follow-up period was 125.6 months (95% confidence interval (CI) 120.3 to 131.0). A total of 106 (38.1%) patients died, and 136 (48.9%) had a recurrence of breast cancer.

**Table 1:** Patient age and histopathological characteristics of 278 breast carcinomas

Characteristic	Number of patients		
	Node-positive, pN1–3	Node-negative, pN0	pNx
	*n *= 131	*n *= 137	*n *= 10
pT			
pT1	32	64	3
pT2	56	66	4
pT3	14	3	1
pT4	29	1	2
pTx	-	3	-
Histological grade			
G1	14	22	3
G2	52	75	5
G3	69	36	2
Gx	-	4	-
ER status			
0	26	26	5
1–12	79	72	5
ERx	26	39	-

The median age of patients was 55 years (range 25 to 82 years).

### Tissue microarray (TMA) construction

TMAs were prepared as described previously [24,25]. For each tumour a representative tumour section was selected from a haematoxylin/eosin-stained section of the donor block. Core cylinders with a diameter of 1.5 mm each were punched from this area with a thin-walled stainless steel tube and deposited into a recipient paraffin block. TMA sections were mounted on charged slides (SuperFrost™Plus; Menzel GmbH, Braunschweig, Germany). Haematoxylin/eosin-stained TMA sections were used for reference histology.

### Fluorescence *in situ *hybridisation

FISH was performed on 5 μm sections of the TMAs with the use of directly labelled DNA probes for *HER1*, *HER2*, *HER3 *and *HER4 *(ZytoVision Ltd., Bremerhaven, Germany). The probes identified locus-specific sequences for both the genes and the corresponding centromeres 7, 17, 12 and 2 to differentiate between gene amplification and polysomy of the respective chromosome.

TMA sections were dewaxed for 40 minutes in an incubator at 72°C and twice for 10 minutes in xylene. After being rehydrated in a graded ethanol series and rinsed in distilled water, slides were placed in 0.01 M sodium citrate and steamed for 40 minutes in a water bath. Cell structures were digested in 0.1% pepsin (Sigma, Munich, Germany) and 0.01 M HCl for 10 minutes at 37°C. After washing in 2 × SSC (1 × SSC (standard saline citrate) is 150 mM sodium chloride and 15 mM sodium citrate, pH 7) and water, slides were dehydrated in graded alcohols and air-dried. Respective DNA probe sets (10 μl each) were applied to the TMA area of each section. Sections were coverslipped and the edges were sealed with rubber cement. For co-denaturation of the probe and target DNA, slides were placed for 5 minutes on a hotplate preheated to 73°C and than transferred overnight to a warmed hybridisation chamber at 37°C. After hybridisation, the rubber cement was removed and the slides were immersed successively in 4 × SSC plus 0.3% Igepal (Serva, Heidelberg, Germany), 2 × SSC and 1 × SSC for 10 minutes at 50°C. The slides were rinsed briefly in Millipore water and air-dried. Nuclei were counterstained with anti-fading DAPI (4',6-diamidino-2-phenylindole) Vectashield (Vector Laboratories, Burlingame, CA, USA) and were analysed by epifluorescence microscopy.

### Microscopy, fluorescence *in situ *hybridisation scoring and digital imaging

Slides were imaged with an Axio Imager Z.1 (Zeiss, Göttingen, Germany) equipped with specific filter sets for DAPI fluorescence (excitation 365 ± 20 nm, emission 450 ± 25 nm; Zeiss), green fluorescence (excitation 500 ± 10 nm, emission 535 ± 15 nm) and red fluorescence (excitation 545 ± 15 nm, emission 610 ± 35 nm; AHF, Tübingen, Germany). Fluorescence images were obtained with a Plan-Apochromat lens (63×, numerical aperture 1.4) and recorded with a CCD (charge-coupled device) camera AxioCam MRm (Zeiss). The plug-in module ApoTome™ enabled the taking of pseudoconfocal, scattered out-of-focus light-free images using transmission grids and corresponding algorithms. To exclude the loss of FISH signals, three-dimensional *z*-stacks were generated. Each colour was recorded and digitally processed (filtering and contrast enhancement) using AxioVision 4.5 software (Zeiss). Corresponding images were superimposed.

FISH scoring was performed by counting fluorescence signals in 25 malignant, non-overlapping cell nuclei for each case by two independent interpreters (AS, MB). The FISH ratio was assessed as the number of genes proportional to the number of centromeres.

### Immunohistochemistry

**Figure 1:**
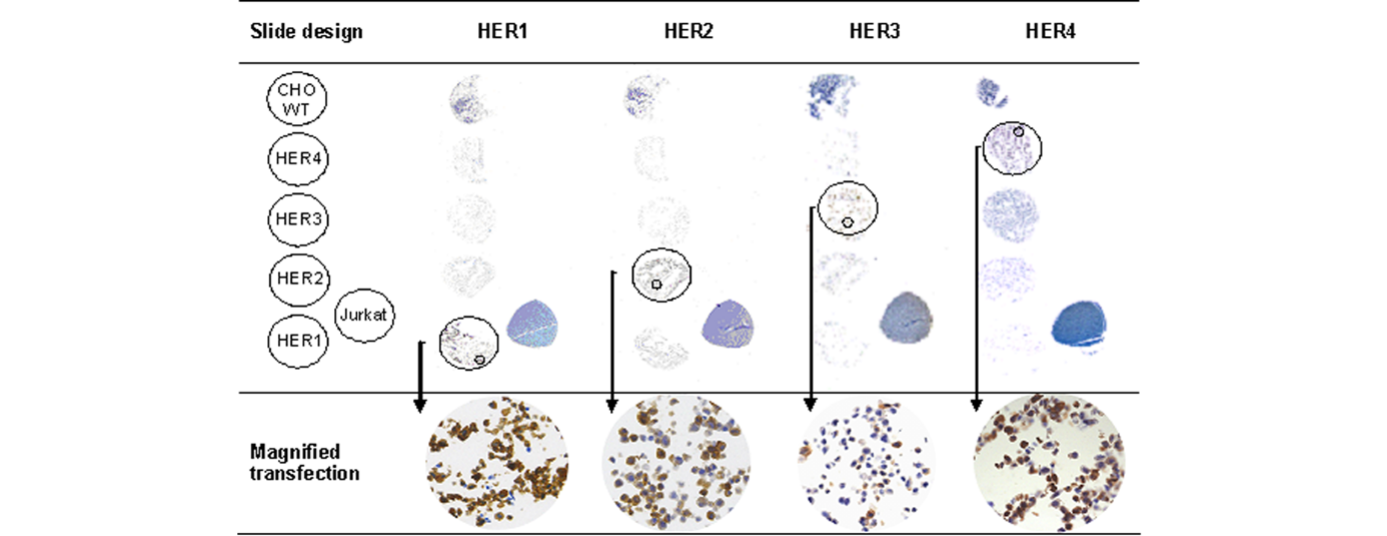
Anti-HER-immunostaining of mouse fibroblasts, stably transfected with *HER1*, *HER2*, *HER3 *or *HER4*. Chinese hamster ovary wild-type, Jurkat and the three additional transfected fibroblast cell lines served as negative controls (magnification of slide overview ×16, magnification of cutout ×400, small circle indicates magnified area).

Immunostaining with anti-HER-receptor antibodies and MIB-1 (anti-Ki-67) was performed on 5 μm sections of the TMAs and applied in accordance with the manufacturer's instructions. Table 2 shows the antibody-specific staining and scoring characteristics. MIB-1 was regarded as positive when 30% or more of the nuclei in the punched tissue were stained. Interpretation was performed independently by two experienced pathologists (SS, AH). Stably transfected mouse fibroblasts proved specific immunostaining (Figure 1).

**Table 2:** Characteristics for anti-HER and anti-Ki-67 immunostaining and scoring

Characteristic	HER1	HER2	HER3	HER4	Ki-67
Antibody	Mouse mAb	Rabbit pAb	Mouse mAb	Rabbit mAb	Mouse mAb
Origin	Dako	Dako	NanoTools	Cell Signaling	Dako
Clone	2–18C9	-	5A12	83B10	MIB-1
Concentration of primary antibody	EGFR pharmDX	HercepTest (2.5 μg/ml)	5 μg/ml culture supernatant	0.7 μg/ml	1.6 μg/ml
Staining pattern	Membrane	Membrane	Cytoplasm and membrane	Cytoplasm and membrane	Nucleus
Epitope retrieval	Proteinase K	Heat induced, 10 mM citric acid buffer, pH 6.0	Heat induced, 10 mM citric acid buffer, pH 7.3	Heat induced, 10 mM sodium-citric acid pH 6.0	Heat induced, 10 mM citric acid buffer, pH 7.2
Blocking	Endogenous peroxidase blocking	Endogenous peroxidase blocking	Endogenous peroxidase blocking	Endogenous peroxidase blocking	Endogenous peroxidase blocking
Primary antibody	Overnight, 4°C	Overnight, 4°C	Overnight, 4°C	Overnight, 4°C	30 min, room temperature
Detection system	EnVision™ Dual Link System (Dako); DAB + chromogenic substrate	EnVision™ Dual Link System (Dako); DAB + chromogenic substrate	EnVision™ Dual Link System (Dako); DAB + chromogenic substrate	EnVision™ Dual Link System (Dako); DAB + chromogenic substrate	*i*VIEW™ DAB Detection Kit (Ventana)
Scoring in accordance with	EGFR pharmDx guidelines	HercepTest guidelines	EGFR pharmDx guidelines	EGFR pharmDx guidelines	Manufacturer's guidelines

mAb, monoclonal antibody; pAb, polyclonal antibody; DAB, diaminobenzidine; EGFR, epidermal growth factor receptor.

### Statistical analyses

The primary outcome measure, overall survival, was calculated as the time from the date of diagnosis to death from any cause or the date on which the patient was last known to be alive. Patients lost to follow-up were treated as censored cases on the basis of the date they were last known to be alive. The secondary outcome measure was the disease-free survival, the time from diagnosis to the date of tumour-related death. Two outcome-orientated approaches were used to determine the cut-off points for *HER1–HER4 *FISH with regard to overall survival. First, we examined plots of the martingale residuals against the single *HER1–HER4 *FISH variables using the PROC LOESS option in SAS and chose DIRECT SMOOTH with a smoothing parameter of 2/3. Second, we applied the Contal and O'Quigley method [26], which is based on the log rank statistic and provides *p *values corrected for examining multiple potential cut-off points. The cut-off points obtained were then used to divide patients into two groups: amplified and non-amplified. Survival curves were generated by using the Kaplan–Meier method, and log-rank tests compared the distributions between groups. In addition, hazard ratios (HR values) with 95% CIs were estimated for a single covariate (treated as continuous, and where appropriate as a dichotomous variable) using the Cox proportional-hazards model. Finally, multivariate Cox models were fitted to assess the prognostic significance of *HER1*, *HER3 *and *HER4 *irrespective of *HER2*. For cut-off point determination an adjusted 10% level of significance was used. In all other analyses, *p *≤ 0.05 (two-tailed) was considered significant. Statistical analyses were performed with SPSS version 13.0 and SAS version 9.1 software (SAS Institute, Cary, NC, USA). For cut-off point determination an SAS macro provided by Mandrekar and colleagues [27,28] was applied.

## Results

Microarrays of paraffin-embedded breast cancer tissue from 278 patients were used to analyse gene amplification and protein expression of each member of the HER family. Furthermore, we analysed tumour and nodal status and tumour grading (Table 3) to indicate the representativeness of our patient collective (*p *< 0.001).

**Table 3:** Cox proportional hazards analysis for overall and disease-free survival

Technique	Parameter	Overall survival			Disease-free survival		
		HR	95% CI	*p*	HR	95% CI	*p*
FISH	*HER1*						
	Continuous 0.1	1.02	0.97–1.07	0.380	1.03	0.98–1.07	0.301
	Continuous 1.0	1.24	0.77–2.00		1.28	0.80–2.03	
	*HER2*						
	Continuous 0.1	1.02	1.01–1.02	0.001	1.02	1.01–1.03	<0.001
	Continuous 1.0	1.16	1.06–1.26		1.18	1.08–1.29	
	Dichotomous (≤1.5 vs. ≥1.6)	2.07	1.33–3.21	0.001	2.33	1.48–3.68	<0.001
	*HER3*						
	Continuous 0.1	1.07	0.99–1.15		1.09	1.01–1.18	0.031
	Continuous 1.0	1.88	0.88–4.01	0.102	2.35	1.08–5.11	
	*HER4*						
	Continuous 0.1	0.90	0.75–1.09	0.289	0.92	0.76–1.12	0.395
	Continuous 1.0	0.36	0.06–2.36		0.43	0.06–3.03	
IHC	HER1	1.66	0.96–2.86	0.070	1.55	0.87–2.77	0.139
	HER2	1.42	0.90–2.25	0.131	1.58	0.99–2.54	0.057
	HER3	0.84	0.51–1.37	0.484	0.93	0.55–1.56	0.778
	HER4	1.48	0.97–2.26	0.070	1.51	0.97–2.33	0.066
	HER2 divided	1.72	1.07–2.75	0.024	1.89	1.16–3.08	0.010
	Ki-67	1.49	0.87–2.55	0.145	1.44	0.83–2.49	0.197
	ER	0.58	0.37–0.91	0.018	0.59	0.37–0.96	0.031
	PR	0.48	0.27–0.74	0.002	0.42	0.24–0.71	0.001
Histology	pT	1.62	1.35–1.93	<0.001	1.72	1.43–2.08	<0.001
	pN	1.98	1.67–2.35	<0.001	2.18	1.81–2.62	<0.001
	Grading	2.10	1.54–2.87	<0.001	2.18	1.56–3.05	<0.001

Hazard ratios (HR), confidence intervals and *p *values of investigated parameters dependent on overall and disease-free survival (CI, confidence interval with lower and upper limits). All immunohistochemical and histological parameters were used as categorical variables. FISH, fluorescence *in situ *hybridisation; IHC, immunohistochemistry.

### Fluorescence *in situ *hybridisation

**Figure 2:**
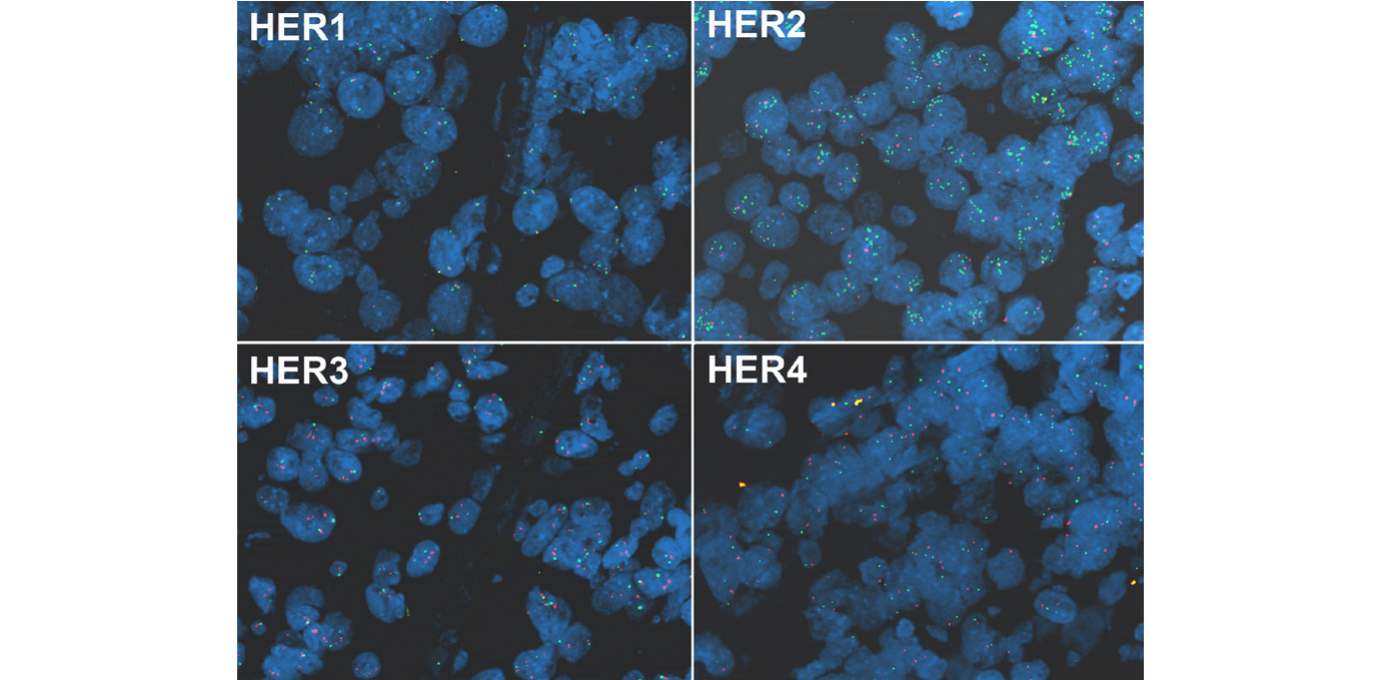
Anti-*HER1–HER4 *FISH in breast cancer tissue of one patient (dual probes). *HER1*: red cen 7, green loc 7p11 (diploid); *HER2*: red cen 17, green loc 17q12 (amplified); *HER3*: green cen 12, red loc 12q13 (moderately amplified); *HER4*: green cen 2, red loc 2q33 (diploid); 4',6-diamidino-2-phenylindole core staining blue. Cen, centromere; loc, gene locus.

Newly designed probes for dual-colour FISH (courtesy of ZytoVision Ltd., Bremerhaven, Germany) were established to detect the gene and the centromere status of each receptor (Figure 2).

**Figure 3:**
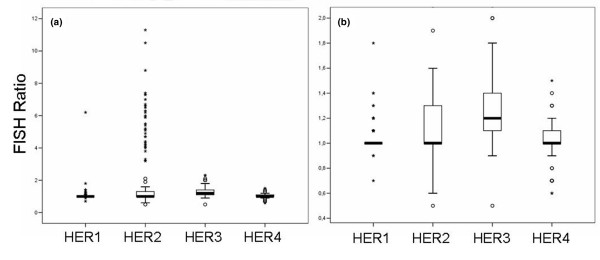
Distribution of *HER1–4 *FISH ratios. **(a) **Boxplots of *HER1–HER4 *FISH ratios (gene/centromere). **(b) **Magnified extract of (a) to demonstrate the different distribution pattern of ratios for each *HER*-family member.

The ratio distributions of *HER1–HER4 *FISH are presented in Figure 3. Whereas *HER2 *ratios varied from 0.5 to 11.3, the study population was more homogeneous with regard to *HER3 *(range 0.5 to 2.3) and *HER4 *(range 0.6 to 1.5) and extremely homogeneous with regard to *HER1 *(range 0.7 to 6.2). Thus, not only the range but also the number of different ratios was restricted (*HER2*, 46; *HER1*, *HER3 *and *HER4*, 10 to 17). For *HER1*, these findings resulted in an accumulation of 90.3% of values at a ratio of 1.0 and 1.1, an extremely high percentage compared with its receptor relatives (*HER2*, 59.3%; *HER3*, 28.1%; *HER4*, 77.7%).

**Figure 4:**
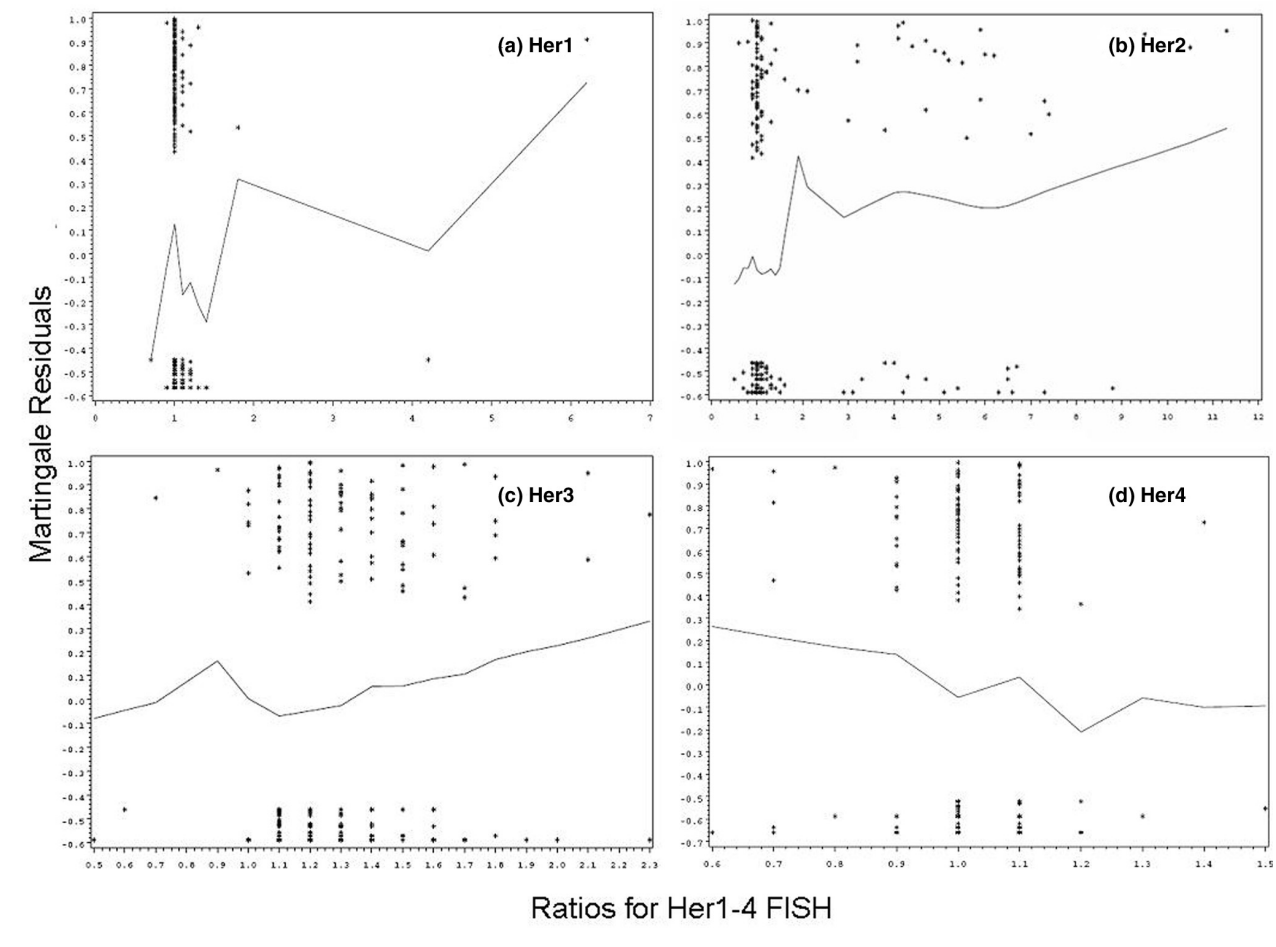
Martingale residuals, plotted against *HER1–HER4 *FISH ratios, based on overall survival. FISH, fluorescence *in situ *hybridisation.

With overall survival as the primary outcome measure, the plots of the martingale residuals versus *HER1–HER4 *FISH (Figure 4) implied the interpretation of *HER1*, *HER3 *and *HER4 *as continuous variables rather than dichotomising the data on the basis of a cut-off point. In contrast, the smoothed curve for *HER2 *FISH was roughly zero up to about 1.5 and then increased rapidly. *HER2 *FISH was therefore converted into a categorical variable. The results from the Contal and O'Quigley method were consistent with the results from the graphical approach. For *HER2 *FISH there were 46 distinct values, any of which could be defined as a potential cut-off point. The most informative value of the log rank statistic occurred at the *HER2 *FISH ratio of 1.5 (adjusted *p *= 0.086). This suggests that the cut-off point obtained is related to overall survival. The patients were therefore divided into two groups: patients with *HER2 *FISH ratios of 1.5 or less, and patients with *HER2 *FISH ratios of at least 1.6. Using the Contal and O'Quigley method for *HER1*, *HER3 *and *HER4 *FISH, again no cut-off point related to overall survival could be defined (adjusted *p *> 0.33). Consequently, in further analyses *HER1*, *HER3 *and *HER4 *FISH were included as continuous variables, whereas *HER2 *FISH was assessed as both continuous and dichotomous variables.

The *HER2 *gene was amplified in most cases without evidence of polysomy, whereas both *HER3 *and *HER4 *gene alterations were usually found in combination with polysomic gene status. This finding partly explains lower FISH ratios for *HER1 *and, in particular, for *HER3 *and *HER4 *compared with *HER2*.

FISH-dichotomised *HER2 *results are presented in Table 4. *HER2 *showed 19.9% (46 of 231) positive cases and 80.1% (185 of 231) negative cases. HR values for continuous distribution of *HER1–HER4 *ratios for single (1.0) and one-tenth (0.1) units are given in Table 3. The HR displays either the increase (HR > 1.0) or the decrease (HR < 1.0) of the risk of mortality by an enlargement of *HER1–HER4 *FISH ratio. A HR value (single unit, 1.0) of 1.16 means that a patient with a *HER2 *FISH ratio of 3.0, for example, has a 1.16-fold higher risk (based on overall survival) than a patient with a ratio of 2.0. Of 46 amplified cases, 40 (87.0%) also overexpressed HER2 protein. A correlation analysis of *HER2 *FISH and immunohistochemistry (IHC; both dichotomous) was positive (*p *< 0.001; correlation coefficient (CC) = 0.809). Reviewing the connection between *HER3 *FISH (continuous) and IHC (dichotomous), we found a significant weak correlation (*p *= 0.038, CC = 0.162) as well as for *HER1 *FISH and IHC (*p *= 0.010, CC = 0.200). *HER4 *FISH and IHC data showed no correlation (*p *= 0.327, CC = -0.075).

**Table 4:** Results of fluorescence *in situ *hybridisation (*HER2*) and immunohistochemical staining (HER1–HER4)

	FISH	Immunohistochemistry			
		
	*HER2*	HER1	HER2	HER3	HER4
Evaluation characteristics	*n *= 231	*n *= 178	*n *= 214	*n *= 173	*n *= 191
0	n.r.	152 (85.4)	112 (52.4)	43 (24.9)	120 (62.8)
1+	n.r.	**7 (3.9)**	54 (25.2)	**115 (66.5)**	**49 (25.7)**
2+	n.r.	**11 (6.2)**	**26 (12.1)**	**14 (8.1)**	**17 (8.9)**
3+	n.r.	**8 (4.5)**	**22 (10.3)**	**1 0.5)**	**5 (2.6)**
Positive	46 (19.9)	26 (14.5)	48 (22.4)	130 (75.1)	71 (37.2)
Negative	185 (80.1)	152 (85.5)	166 (77.6)	43 (24.9)	120 (62.8)

Numbers in parentheses are percentages. The total sample size was *n *= 278. Results in bold define data considered positive for immunohistochemistry. FISH, fluorescence *in situ *hybridisation; n.r. = non-relevant for FISH; cut-off point for *HER2 *FISH analysis (≤1.5 vs. ≥1.6) distinguishes amplified (positive) or normal (negative) gene status.

**Figure 5:**
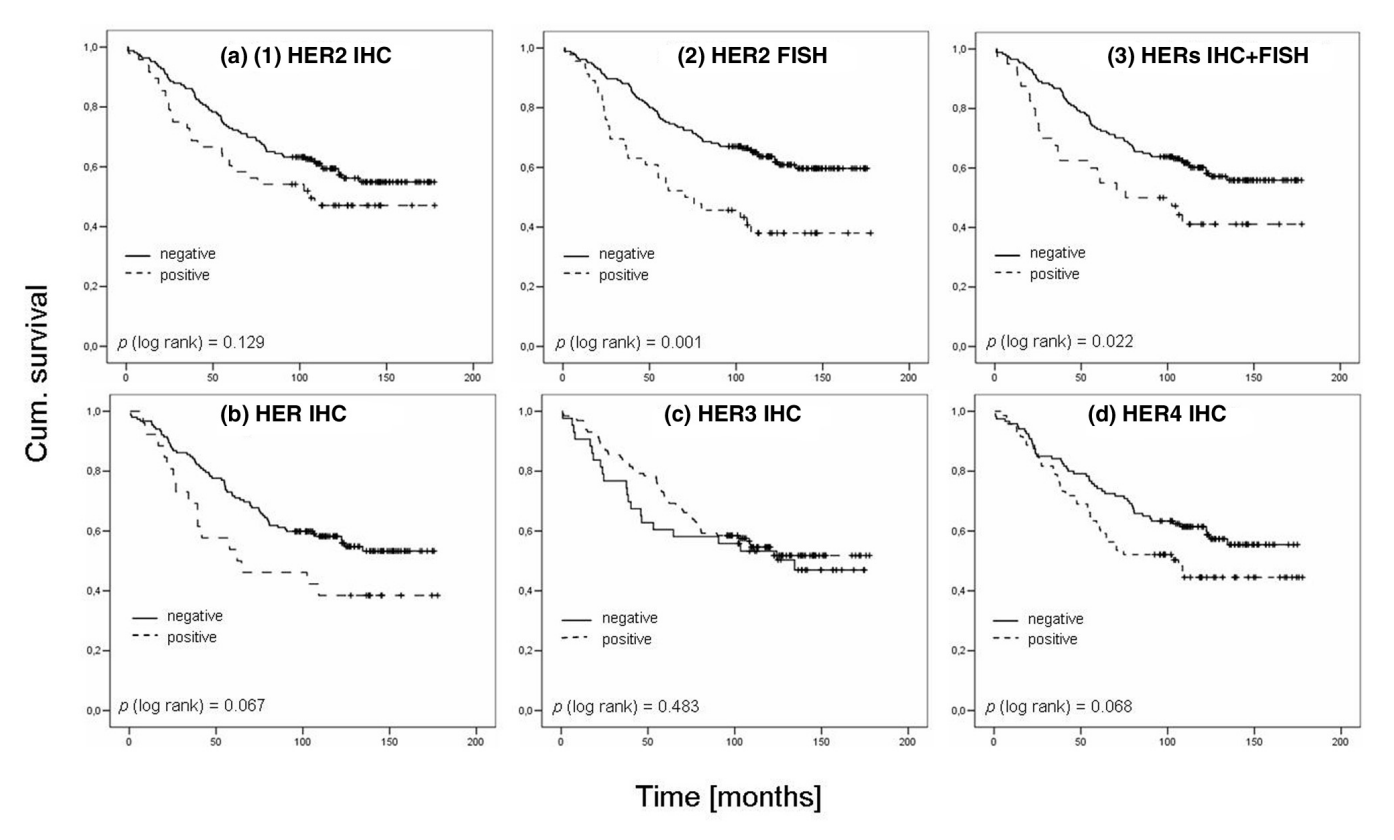
Kaplan–Meier curves of dichotomised variables, based on overall survival. Anti-HER1–HER4 immunohistochemistry **(a1,b,c,d)**, anti-*HER2 *FISH **(a2) **and immunohistochemistry score 2+, stratified by FISH-amplified and non-amplified cases **(a3)**. FISH, fluorescence *in situ *hybridisation.

Patients with *HER2 *amplified breast cancer presented a significantly worse outcome for overall (*HER2 *dichotomised: HR = 2.07 (95% CI 1.33 to 3.21), *p *= 0.001; *HER2 *continuous: HR = 1.16 (95% CI 1.06 to 1.26), *p *= 0.001) and disease-free survival than patients with the non-amplified gene (Table 3 and Figure 5a2).

In addition, *HER3*-positive cases had a shorter disease-free survival (HR = 2.35 (95% CI 1.08 to 5.11), *p *= 0.031). Amplification of *HER1 *caused a negative trend for survival (HR = 1.24 (95% CI 0.77 to 2.00), *p *= 0.380), whereas *HER4 *resulted in a decrease in the HR (HR = 0.36 (95% CI 0.06 to 2.36), *p *= 0.289). For a comparison of overall and disease-free survival values see Table 3.

**Figure 6:**
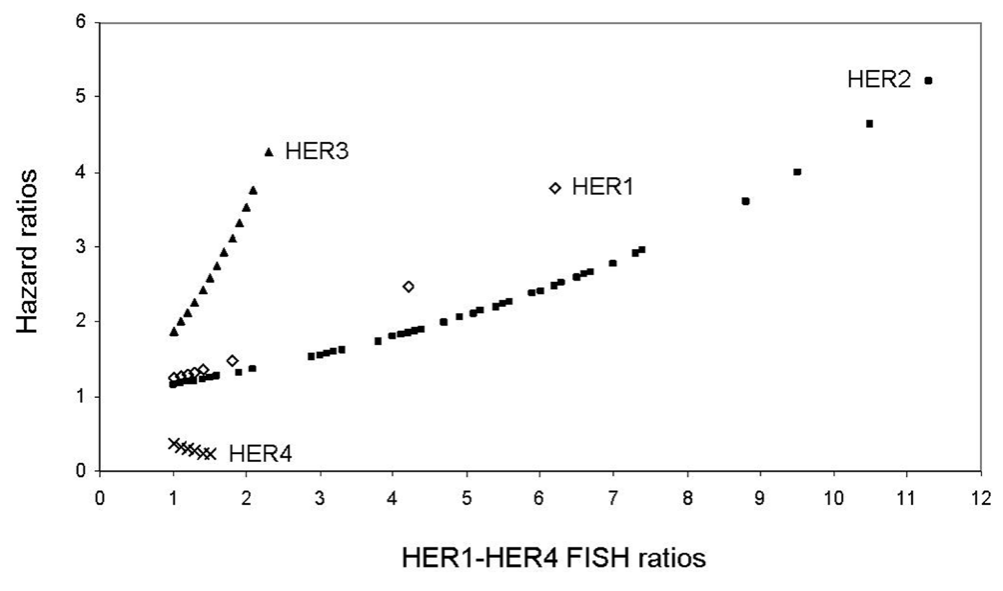
Univariate analysis of *HER1–HER4 *FISH. Comparison of individually calculated hazard ratios (overall survival, HR_ind _= exp [*B*_*HERx *_× *HERx *continuous]), based on the hazard ratio in one-tenth intervals showing the rising or declining hazard level as a function of increasing fluorescence *in situ *hybridisation (FISH) ratio.

After performing univariate analysis based on overall survival, we calculated an individual HR for each patient (HR_ind _= exp [*B*_*HERx *_× *HERx *continuous]) and plotted against the respective receptor gene *HER1 *to *HER4 *(Figure 6). In this analysis, *HER1*, *HER2 *and *HER3 *displayed an increasing HR with raised FISH ratios (continuous), whereas the *HER4 *HR declined. *HER3 *had the steepest curve, followed by *HER1 *and *HER2 *as next steepest.

**Figure 7:**
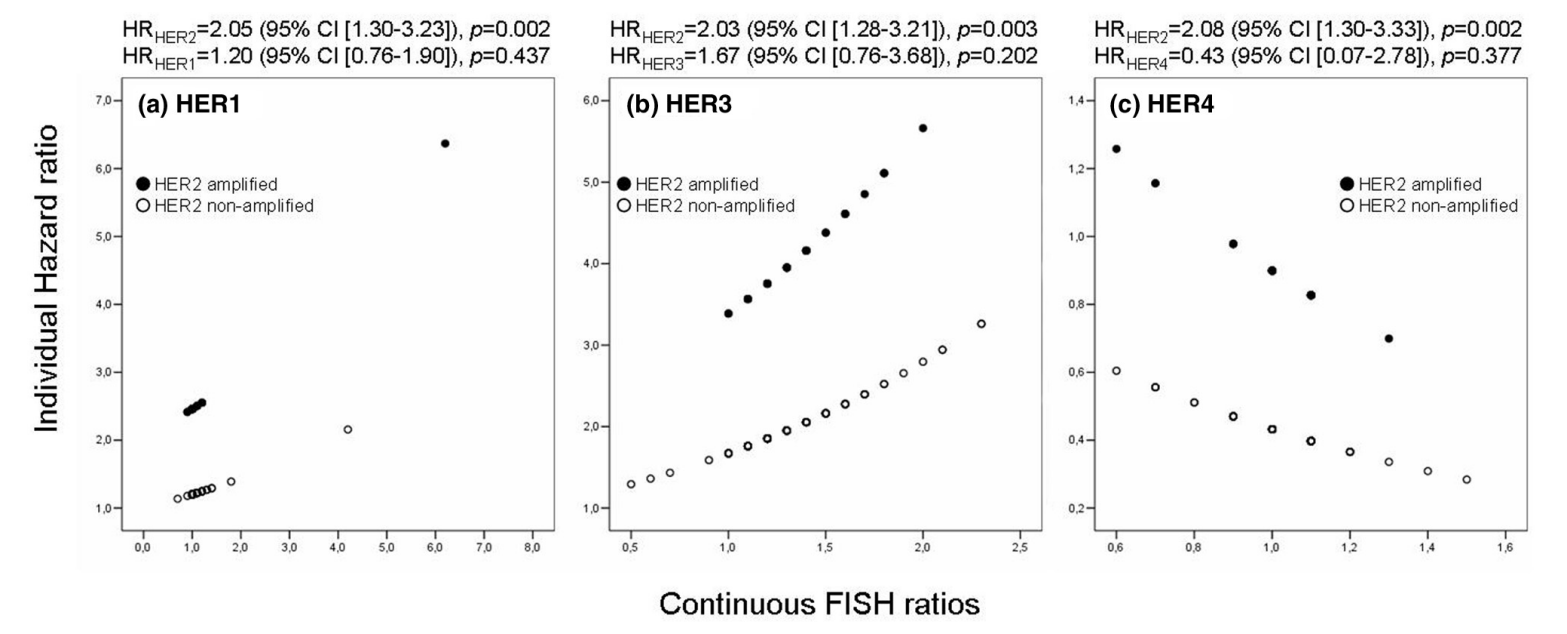
Multivariate analysis of dichotomised *HER2 *FISH with continuous *HER1*, *HER3 *or *HER4 *FISH. Individually calculated hazard ratios (overall survival, HRind = EXP [*B*_*HER*2 _× *HER2 *dichotomised + *B*_*HERx *_× *HERx *continuous]) for *HER1 ***(a)**, *HER3 ***(b) **and *HER4 ***(c) **fluorescence *in situ *hybridisation, divided into *HER2 *amplified (ratio ≥ 1.6; filled circles) and *HER2 *non-amplified (ratio ≤ 1.5; open circles) patients.

Exploring the effects of *HER1*, *HER3 *and *HER4 *in coexpression with *HER2*, *HER2 *results were separated into amplified and non-amplified cases (Figure 7). In a multivariate approach, dichotomised *HER2 *and continuous *HER1*, *HER3 *or *HER4 *were analysed. No significant interaction between *HER2 *and each of its three relatives was found. Thus, for each patient the individual HR without interaction was calculated (HR_ind _= exp [*B*_*HER*2 _× *HER2 *dichotomised + *B*_*HERx *_× *HERx *continuous]). Amplified *HER2 *curves extend beyond non-amplified curves in every case, showing the greater impact of *HER2 *in multivariate Cox regression, visualised by adjusted *HER2 *HR values (Figure 7). *HER1*, *HER3 *and *HER4 *have additional relevance on the basis of increasing FISH ratio, given that the curves do not run parallel to the *x*-axis. Whereas the upper graph displays the impact of *HER2 *amplification dependent on increasing supplemental aberration of a second receptor, the lower curve demonstrates the exclusive impact of this receptor gene irrespective of *HER2*.

Figure 7 suggests that the main effects are additive in nature. Figure 7a,b shows the same monotonically increasing trend: higher *HER1 *and *HER3 *values were associated with higher risk of mortality. This was valid for patients both with and without *HER2 *gene amplification. In contrast, Figure 7c (*HER4*) showed a monotonically decreasing trend: higher *HER4 *values were associated with a lower risk of mortality applied to both the *HER2 *amplified and the *HER2 *non-amplified patient group.

### Immunohistochemistry

Immunostaining of all four HER receptors was performed (Figure 1). The specificity of applied antibodies was proved by staining stably transfected mouse fibroblasts (NIH 3T3, kindly provided by Roche Diagnostics, Penzberg, Germany).

For IHC, 14.5% (26 of 178) were identified as HER1 positive (score 1+, 2+ and 3+), and 85.5% (152 of 178) as negative (Table 4). In 22.4% (48 of 214) HER2 was overexpressed (score 2+ and 3+), and 77.6% (166 of 214) were normal. IHC of HER3 resulted in 75.1% (130 of 173) positive (score 1+, 2+ and 3+) and 24.9% (43 of 173) negative patients. For HER4, 37.2% (71 of 191) of cases presented overexpression (score 1+, 2+ and 3+), whereas 62.8% (120 of 191) did not.

Overexpressed HER2 receptors (Figure 5a1) were associated with decreased overall survival (*p *= 0.129; HR = 1.42 (95% CI 0.90 to 2.25), *p *= 0.131), a well established observation that became unambiguously evident in cases that were scored IHC 2+ and *HER2 *FISH positive (Figure 5a3; *p *= 0.022; HR = 1.72 (95% CI 1.07 to 2.75), *p *= 0.024). A comparison of positive and negative HER3 cases (Figure 5c) did not yield any supplemental information (*p *= 0.483; HR = 0.84 (95% CI 0.51 to 1.37), *p *= 0.484). Immunostaining of HER1 (Figure 5b) indicated a negative effect for patients with overexpressed levels (*p *= 0.067; HR = 1.66 (95% CI 0.96 to 2.86), *p *= 0.070). In addition, HER4 protein overexpression (Figure 5d) tended to have a negative impact on disease (*p *= 0.068; HR = 1.48 (95% CI 0.97 to 2.26), *p *= 0.070).

To examine the proliferation status in our patient cohort we assessed Ki-67 IHC. In 24% of cases (36 of 150) we documented a positive staining (more than 30% of nuclei) with a negative effect on overall survival, whereas 76% of cases (114 of 150) showed less or no staining resulting in a more favourable outcome (*p *= 0.142; HR = 1.49 (95% CI 0.87 to 2.55), *p *= 0.145).

## Discussion

Because screening of HER2 aberration as a prerequisite for Herceptin therapy [29-31] enables the prediction of neither the course of disease nor the individual response, the identification of additional prognostic and predictive parameters is of the utmost interest, primarily being those with immediate impact on HER2. Further investigation of the additional three, highly homologous HER2 cognate members of the human EGFR (HER-) tyrosine kinases [32] is well founded. Here we present first-hand data of a four-target FISH and IHC analysis comprising all HER receptors in breast carcinomas by using TMA. The aim of this study was to identify HER2-related molecules with additional prognostic significance within the HER family.

We verified the known negative impact of *HER2 *amplification on the overall and disease-free survival of patients. The discrepancy of 13% of the patients showing amplification of the *HER2 *gene but not overexpression of the HER2 protein is consistent with the expected loss of IHC sensitivity associated with tissue fixation and embedding [33]. The positive correlation between *HER2 *FISH and HER2 IHC analysis is in accordance with the literature (up to 95% concordance between FISH and IHC) [2,6] as well as our own previous data (100% concordance between FISH and fluorescent IHC) [34].

Similar to our FISH and IHC analysis, Tsutsui and colleagues [19] found the combination of HER1 and HER2 expression in breast cancer to have a severe negative impact on disease outcome compared with normal protein levels, whereas the prognostic value of HER2 overexpression seemed more pronounced than HER1 overexpression. However, Diermeier and colleagues [16] provided evidence that the HER1 expression level in the breast cancer cell line SK-BR-3, coexpressed with overexpressed HER2, has a key role in mediating the anti-proliferative effect of Herceptin. Overall, *HER1 *gene amplification or HER1 protein overexpression in this study was found to be a rare event.

In contrast, the data presented in this study provide striking evidence for a significance of alterations in HER3 in breast cancer. This observation is supported by Holbro and colleagues [35], who identified the function of the HER2/HER3 dimer as an oncogenic unit in which HER3 couples active HER2 to the downstream signalling phosphoinositide 3-kinase/protein kinase B pathway. Blocking HER2 resulted in antiproliferative effects accompanied by a decrease in HER3 signalling activity [36]. Although HER3 has no intrinsic kinase activity to initiate the signalling process, ligand-bound or even ligand-independent HER3 may form heterodimers with HER2 that are potent signalling complexes [37,38]. According to Liu and colleagues [39], HER3 also contributes to HER2-associated tamoxifen resistance, and a decrease in HER3 levels restores sensitivity to tamoxifen. Jones and colleagues [40] provided a quantitative protein interaction network by applying protein microarrays comprising virtually every Src homology 2 (SH2) and phosphotyrosine-binding (PTB) domain encoded in the human genome. They found a difference in the extent to which the HER receptors form protein–protein interactions when overexpressed, and consequently found the HER2–HER3 complex to have the most pronounced promiscuity with regard to activate intracellular signalling. Our results indicate the significance of the *HER2*/*HER3 *aberration in the increasing HR and therefore risk of mortality after multivariate analysis of *HER2 *and *HER3 *(Figure 7).

We detected a positive impact of *HER4 *on disease outcome with FISH but not with IHC analysis, as reflected by the lack of correlation between FISH and IHC data. HER4 protein overexpression has previously been described as a positive prognostic factor, a suggestion based on investigative approaches [20,41,42]. These positive effects can most probably be attributed to growth controlling and differentiation signalling. Barnes and colleagues [43] showed that HER4 decreases HER2 signalling activity by leading to decreased proliferation activity and increased apoptosis. In accordance with these data, we were also able to show via FISH analysis a *HER2*-compensating effect of *HER4 *represented by a decreased *HER2 *HR in the presence of alteration of *HER4 *(Figure 7). Furthermore, in support of a potential impact of *HER4 *amplification on improved outcome, we found that *HER4 *amplification detected by FISH was correlated with a positive oestrogen receptor status (*p *= 0.001, CC = 0.266) [18,44,45]. Significant correlation of HER4 positivity with low bromodeoxyuridine-derived proliferation indices as described by Tovey and colleagues [18] is associated with a good prognosis in breast cancer tumours. Contrary conclusions were reported by Vogt and colleagues [46], who found that *HER4 *amplification and ER activity were negatively correlated. These differences may occur as a result of the variable responses by HER4 to its activating ligand Heregulin, resulting in either proliferation or differentiation, and perhaps influenced by homodimerisation or heterodimerisation with other HER-family members [47].

Vidal and colleagues considered that the HER4 cell-killing intracellular domain 4ICD might be responsible for association with overall improved patient prognosis [48] by accumulating in mitochondria, causing an efflux of cytochrome *c *and resulting in mitochondrion-regulated apoptosis [49]. It is known that HER-receptor activity and signalling is variable and depends on a particular receptor coexpression profile, potentially explaining the unequivocally strong correlation between HER4 alteration/overexpression and tumour grade or proliferation index [50]. To discover a potential relationship between the proliferation index and *HER4 *FISH-associated positive or HER4 IHC-associated negative patient outcome, we performed Ki-67 immunostaining. Positive MIB-1 IHC resulted in a negative trend (overall survival) in a univariate analysis, which is consistent with the literature [21]. In fact, Her4 IHC and Ki-67 were positively correlated (*p *= 0.002, CC = 0.256), whereas no correlation was found between *HER4 *FISH and Ki-67 (*p *= 0.267, CC = -0.095). Detailed functional studies addressing the impact of HER4 in the context of well-described coexpression patterns will elucidate the importance of HER4 within the HER-receptor family.

Information on HER1, HER3 and HER4 protein overexpression is extremely variable in the literature [20,44,51]; for example 16 to 36% for HER1, 18 to 26% for HER3 and 12 to 82% for HER4, but is usually similar for HER2 (23 to 27%). Our data fell within these ranges, except for HER3 IHC results (75%). With regard to a HER2 coexpression profile, 18.4% of cases additionally overexpressed HER1, 85.0% HER3 and 71.4% HER4. Although the protein statuses of HER2 and HER3 (*p *= 0.013, CC = 0.197) and HER2 and HER4 (*p *< 0.001, CC = 0.446) were significantly correlated with one another, no such correlation was found between HER2 and HER1 IHC (*p *= 0.654, CC = 0.035), an observation supported by the results of Hudelist and colleagues [41].

Overall, the quality of immunohistochemical studies seems to be highly inconsistent because of several factors such as individual tissue preparation, the application of different detection antibodies with different binding specificity, and user-dependent interpretation of staining pattern and intensity [52]. Hence, as demonstrated for HER2, in contrast to FISH, IHC is most probably the less reliable tool for discriminating patients on the basis of alterations in HER receptors [33,53]. Particularly with regard to HER3 and HER4, numerous antibodies are commercially available from which we could prove only one to be specific for each receptor (Figure 1). Furthermore, HER-receptor overexpression can change during breast cancer development, and both a decrease and an increase in expression have been observed [42,54], additionally challenging the interpretation of staining results.

In addition, the prognostic value of HER-family mRNA expression has been a matter of controversy. Bieche and colleagues [55], using real-time quantitative RT-PCR in patients with known long-term survival, found *HER1 *to be underexpressed in 82.3% of cases, *HER2 *(16.9%) and *HER3 *(46.2%) to overexpressed and *HER4 *both underexpressed (29.2%) and overexpressed (24.6%). Among patients with high *HER4 *mRNA levels, a shorter recurrence-free survival was found, suggesting that *HER4 *mRNA status might reflect a marker of poor outcome. However, Zaczek and colleagues [56] recently linked *HER4 *amplification (differential-display PCR) to favourable characteristics, as well as higher levels of *HER3*. RNA–RNA *in situ *hybridisation might clarify any discrepancy between gene and protein states and might fill an information gap.

Further subdivision of the patient cohort into subgroups with regard to individual patient treatment or additional clinical parameters was not considered in the study presented here but should be investigated in a larger patient cohort to examine the predictive value of HER1 to HER4.

## Conclusion

FISH with hybridisation probes targeted to all genes encoding HER receptors turned out to be a sensitive and reliable tool for detecting potential alterations in breast cancer. Although the dominant importance of HER2 over other HER receptors is globally accepted [19], we were able to show a substantial impact of *HER3 *amplification on outcome of breast cancer disease (disease-free survival) even at low amplification rates. Our data provide initial evidence for the integration of *HER3 *as well as *HER4 *analysis into the diagnosis of breast cancer. Investigations are currently under way to determine the clinical importance of individual but interrelated alterations in HER in breast cancer at both the gene and protein levels. An integrated quantification of individual patterns of HER-receptor alterations may enable optimised patient stratification with respect to disease outcome. The quantification of the activated receptor relative to the unactivated protein is a promising approach, particularly with regard to therapeutic response. Further descriptive and functional studies of HER receptors will serve to characterise the disease in terms of a given molecular HER-receptor equivalent, thus providing an essential basis for individualised therapy.

## Abbreviations

B = regression coefficient β; CC = correlation coefficient; CI = confidence interval; DAPI = 4',6-diamidino-2-phenylindole; EGFR = epidermal growth factor receptor; FISH = fluorescence *in situ *hybridisation; HER = human EGFR related; HR = hazard ratio; IHC = immunohistochemistry; RTK = receptor tyrosine kinase; SSC = standard saline citrate; TMA = tissue microarray.

## Competing interests

The authors declare that they have no competing interests.

## Authors' contributions

AS wrote the manuscript, performed the image analysis and FISH evaluation and analysed the statistical data. JR analysed the statistical data and helped to write the manuscript. PJW performed the histological analysis. AH performed the histological analysis and interpreted the data. FH provided material to be analysed and performed primary tissue based diagnostics. SS was a co-senior author, performed the histological analysis, FISH analysis and interpreted the data. GB is the research group leader and senior author and evaluated and interpreted the data.
